# The relationship between serum hepatitis B virus DNA level and liver histology in patients with chronic HBV infection

**DOI:** 10.1371/journal.pone.0206060

**Published:** 2018-11-07

**Authors:** Changjiang Liu, Li Wang, Huizhong Xie, Liyuan Zhang, Bingshu Wang, Chun Luo, Suiqun Wang, Mingliang Tang, Zhongbiao Fu, Hailan Ruan, Zhengjin Liu, Ling Wei, Wenyi Yi, Yunqian Xie

**Affiliations:** 1 Department of Gastroenterology, The Second Affiliated Hospital of Hainan Medical University, Haikou, Hainan Province, China; 2 Department of Pathology, The Second Affiliated Hospital of Hainan Medical University, Haikou, Hainan Province, China; 3 Department of Infectious Diseases, The Second Affiliated Hospital of Hainan Medical University, Haikou, Hainan Province, China; Medizinische Fakultat der RWTH Aachen, GERMANY

## Abstract

**Background:**

There is no consensus regarding the relationship between HBV DNA and liver fibrosis, and the relationship between HBV DNA and the degree of liver cirrhosis has not been reported in patients with chronic HBV infection.

**Methods:**

From January 2011 to December 2016, liver biopsies were performed on 396 patients with chronic hepatitis B and cirrhosis. Assessments of liver fibrosis and cirrhosis were based on the Laennec staging system.

**Results:**

Serum levels of HBV DNA were correlated with fibrosis and cirrhosis (KW = 73.946, P<0.001). Serum HBV DNA level was correlated with mild fibrosis, moderate to severe fibrosis and cirrhosis (P = 0.009, P<0.001, and P<0.001, respectively). The HBeAg-positive group and HBeAg-negative group showed significant differences in HBV DNA levels, and the rates of mild fibrosis, severe fibrosis and cirrhosis were significantly different between these two groups (F = 17.585, P<0.001 and F = 6.017, P = 0.003, respectively). The replication status of the serum HBV DNA affected fibrosis formation as well as cirrhosis (χ^2^ = 53.76, P<0.001). In the HBeAg-positive group, the sensitivity, specificity and AUC values of HBV DNA as a predictor for mild fibrosis and cirrhosis were 64.3%, 78.94% and 0.818, respectively, and 81.0%, 69.2%, and 0.871, respectively. In the HBeAg-negative group, the sensitivity, specificity and AUC values of HBV DNA for liver sclerosis prediction were 48%, 76.8% and 0.697, respectively.

**Conclusions:**

Different HBV DNA levels had different effects on the formation of fibrosis and sclerosis in liver tissues. HBV DNA levels can predict mild fibrosis and cirrhosis in liver tissue, which is enhanced in HBeAg-positive patients.

## Introduction

Hepatitis B virus (HBV) infection can manifest as different clinical types, including infection with Hepatitis B surface antigen (HBsAg) positivity and non-clinical symptoms, chronic Hepatitis B, cirrhosis and liver cancer [1]. During disease progression, HBV infection can be characterized by different clinical types, fibrosis and pathological changes in the liver tissue. Elucidation of the pathological changes of liver tissue in these patients is important in determining patient treatment plans and assessing their prognosis. At present, the observation of pathological changes in the liver tissue is the gold standard for evaluating fibrosis and cirrhosis of liver tissues in HBV-related liver diseases [2]. There are many other ways to assess the degree of liver fibrosis, such as FibroScan; however, this instrument has not been popularized in China, especially in economically underdeveloped areas, because of its high cost. Serological determination of HBV DNA level is simple and can avoid the trauma of biopsy and the limitation of tissue size. This method is useful to evaluate and predict the degree of liver fibrosis and cirrhosis based on the different levels of HBV DNA in clinical practice [3]. The aims of this prospective study were to confirm the correlation between HBV DNA level and liver histology in 396 patients with HBV-related liver disease and provide a theoretical basis for clinical treatment and disease course assessment.

## Materials and methods

### Study population

**Fig 1:**
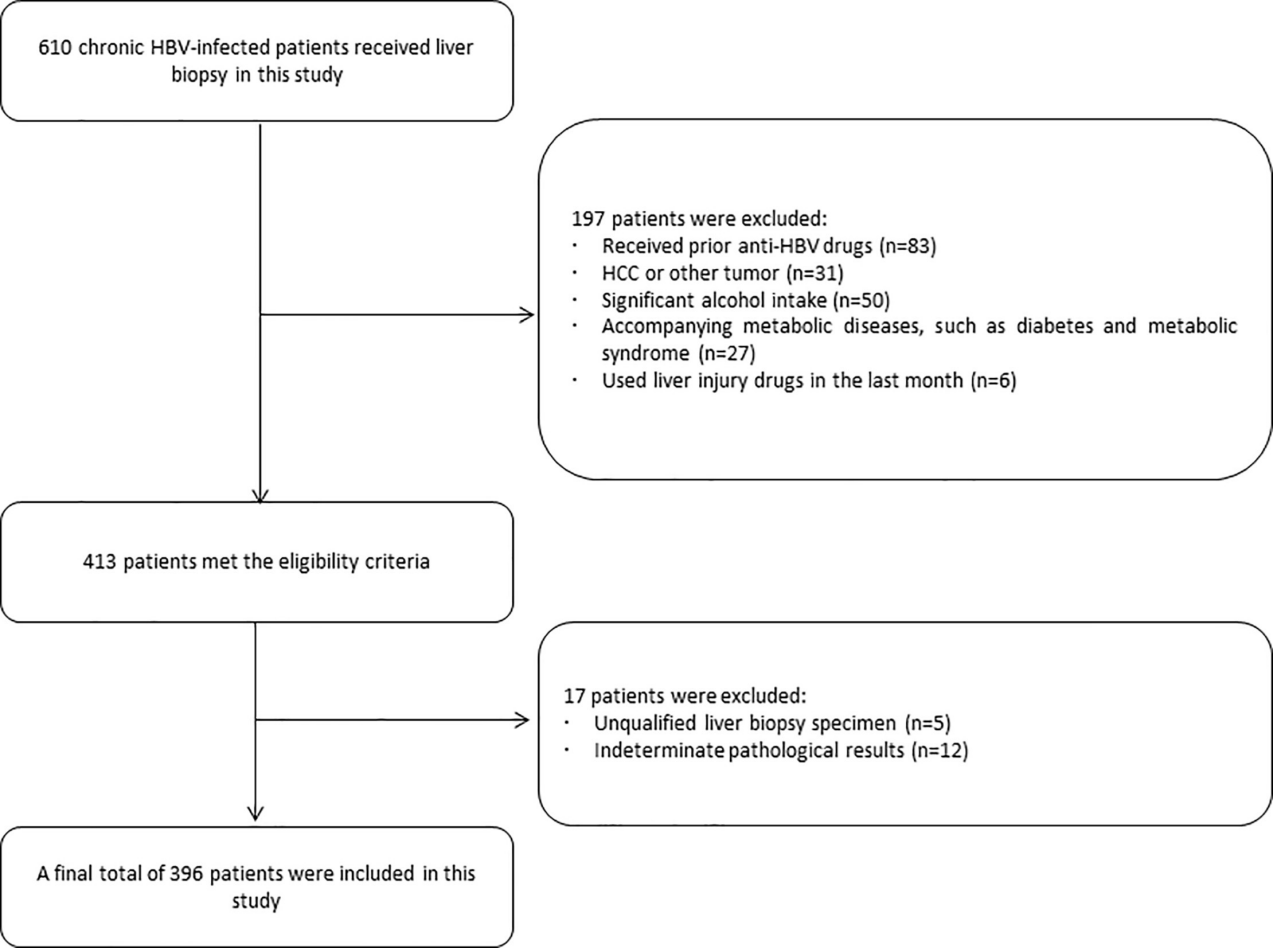
Flow diagram of the study population selection.

From January 2011 to December 2016, a total of 610 patients with HBV-related liver diseases (including chronic hepatitis B and cirrhosis) who underwent liver biopsy were included in the study. The patients were all hospitalized in the Department of Gastroenterology, Department of Infectious Diseases or Department of Hepatobiliary Surgery. Demographic characteristics and laboratory data including age, sex, alanine aminotransferase (ALT) level, platelet count (PLT), HBeAg status and HBV DNA level were collected at the time of liver biopsy. All patients were HBsAg-positive for at least 6 months before enrolling in the study. Overall, 197 patients were excluded based on exclusion criteria, 17 patients were excluded due to unqualified liver biopsy samples and indeterminate pathological results. The flow diagram of the study population is shown in Fig 1. A final total of 396 HBV-related patients were enrolled in this study, with 287 males and 109 females. Their mean age was 49.62±13.56 years, and the age ranged from 20 to 80 years. The patients were diagnosed according to the EASL 2017 standard [4]. All participants provided written informed consent, and ethical approval for these studies was obtained from the Human Research Ethics Committee of the Second Affiliated Hospital of Hainan Medical University, China.

### Serological HBV DNA assay

Serum ALT was detected with a Hitachi 7600 automatic biochemistry analyzer.

PLT was detected with a Sysmex-1800 automated hematology analyzer with five classification levels. Quantification of serum HBV DNA was performed by real-time fluorescence quantitative polymerase chain reaction (PCR). The instrument was an FO-33A PCR amplification instrument from Hangzhou Bori Technology Co., Ltd. The kits were purchased from Shanghai Renaissance Medical Science and Technology Development Corporation. The specimen collection, preservation and detection process were carried out according to the instructions of the reagents. The detection range was 2.24–8.24 log IU/ml (1x10^2^-1x10^9^ copies/ml). Based on the replication degree of HBV DNA, samples were divided into three groups. HBV DNA <2.24 log IU/ml was the no replication group, 2.24≤HBV DNA<4.24 log IU/ml was the low replication group, and HBV DNA≥4.24 log IU/ml was the high replication group [5].

### Liver biopsy

A Madison SA-6000C color Doppler ultrasound instrument was used. The puncture device was a convex vibration probe, with a lateral direction needle on an externally fixed puncture frame. The biopsy gun was a 16G, 20-cm-long disposable Bud puncture needle with a range of 22 mm. The patients were positioned on the left side, and the correct puncture entry point and path were selected after ultrasound localization. After skin disinfection and local anesthesia, the probe angle was fixed when the screen showed a clear target. A total of three liver tissues were obtained. All 396 liver biopsies were performed in the same B ultrasonic room of the hospital by the same ultrasound doctor. The needle was withdrawn rapidly, and a bandage was used for compression hemostasis. The liver tissues were sent to the pathology department for pathological observation.

### Histopathological assessment

The liver biopsy specimens were stained with hematoxylin-eosin (HE). The assessment of liver fibrosis and cirrhosis was performed at low magnification by three senior pathologists unaware of the clinical and virological results. According to the Laennec staging system (grades L0-L4C), liver fibrosis and cirrhosis were divided into 7 grades [6–9]. If there were different opinions on the stage of the liver biopsy, the patient was excluded.

### Statistical analysis

Statistical analyses were performed using SPSS software version 20.0 (SPSS Inc., Chicago, IL, United States). Due to the non-normal distribution of the HBV-DNA levels, these results are expressed logarithmically. The normally distributed variables are shown as the mean and standard deviation, and the groups were compared by analysis of variance. The rank sum test was used for the comparison between groups with non-normally distributed data. Numerical data were analyzed with the χ^2^ test. The correlation between the Laennec staging system and HBV DNA levels was analyzed with Spearman’s correlation. The influence of clinical indicators on liver fibrosis and cirrhosis was analyzed by multivariate logistic regression. The differences in HBV DNA levels in liver tissues of different pathological grades were compared with single factor variance analysis. The predictive value of HBV DNA level for liver fibrosis and cirrhosis was analyzed by ROC curve analysis. A *P* value of less than 0.05 was considered statistically significant.

## Results

### HBV DNA quantification and liver fibrosis and cirrhosis

The 396 liver tissue samples were divided into 7 groups based on the diagnostic criteria of the Laennec staging system. Age, sex, HBV DNA, PLT and ALT levels were compared among the seven groups. The results showed that there was no significant difference in sex among the groups, but the differences in other clinical indicators among the seven groups were statistically significant (P<0.001). The results are shown in Table 1.

**Table 1:** Correlation analysis between Laennec staging and clinical indicators.

Laennec staging	age	n	Sex (male/female)	HBV DNA (log IU/ml)	ALT (U/L)	PLT (10^9^/L)
L0	38.86±13.25	21	14/7	5.53±1.97	149.38±112.69	182.91±72.35
L1	40.83±13.12	40	26/14	5.80±1.99	171.05±161.09	160.33±47.19
L2	49.19±14.62	75	46/29	5.08±1.92	150.67±111.63	150.75±58.20
L3	48.13±13.66	89	63/26	4.62±1.59	132.03±106.65	133.24±64.52
L4A	52.81±11.86	94	77/17	3.69±1.55	64.95±49.55	118.94±43.74
L4B	54.36±9.52	44	34/10	3.61±1.49	63.27±44.08	117.02±55.69
L4C	56.7±10.86	33	27/6	3.18±1.26	62.97±44.52	97.33±35.52
F/χ^2^	47.38		12.42	73.95	79.75	67.75
*P* value	<0.001		0.06	< 0.001	< 0.001	< 0.001

Laennec staging: L0 is normal, L1-L3 indicate liver fibrosis, L4A -L4C indicate cirrhosis.

### Pathological diagnosis of liver fibrosis and cirrhosis

**Fig 2:**
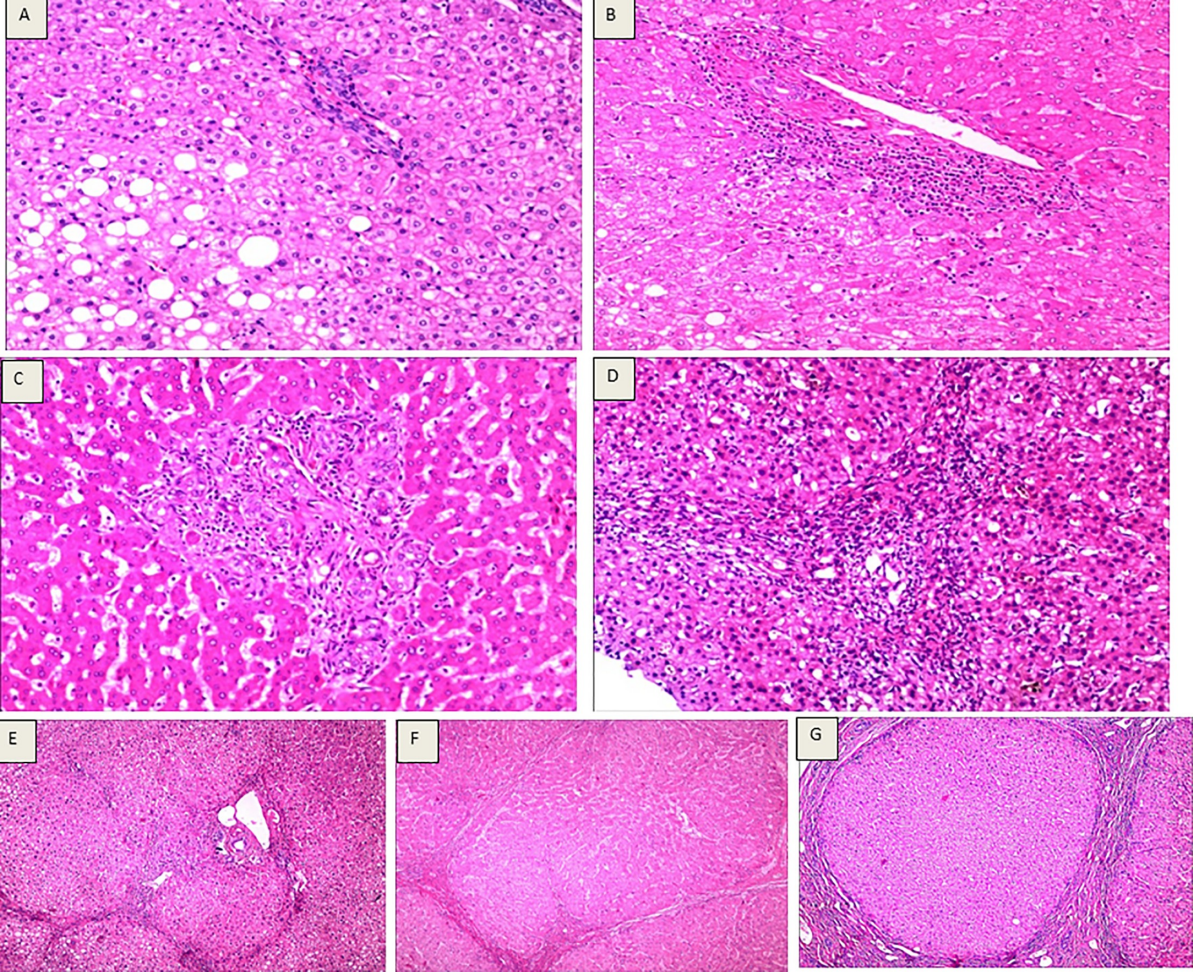
Assessments of liver fibrosis and cirrhosis according to the Laennec staging system. (A) Laennec staging 0, HE staining (10×10), hepatocytic loosening, steatosis, focal necrosis, mild lymphocytic infiltration in the portal area, and no fibrosis. (B) Laennec staging 1, HE staining (4×10), loose liver cell cytoplasm, fatty degeneration, focal point necrosis, mild fiber hyperplasia and enlargement, no fiber spacing, lymphocyte infiltration within the portal area. (C) Laennec staging 2, HE staining (4×10), hepatocyte eosinophilic degeneration, eosinophilic body formation, focal necrosis, enlargement of fibrous tissue in the portal area, formation of fibrous septa, infiltration of lymphocytes, and hyperplasia of small bile ducts. (D) Laennec staging 3, HE staining (4×10), liver cell cytoplasm of osteoporosis, eosinophilic degeneration, cholestasis, focal necrosis and severe periportal piecemeal necrosis, hyperplasia of fibrous tissue expansion, formation of fibrous septa, segmentation of hepatic lobule, periportal and septa lymphocytes. (E) Laennec staging 4A, HE staining (4×10). Hepatic lobule structure disappearance, hepatocyte loosening, steatosis, focal necrosis, fibrous tissue hyperplasia, fibrous septum, formation of false lobules, infiltration of lymphocytes in the portal area and intercellular septum. (F) Laennec staging 4B, HE staining (4×10), hepatic lobule structure disappearance, liver cell vacuolization, focal necrosis and moderate periportal piecemeal necrosis, hyperplasia of fibrous tissue formation of fibrous septa, pseudolobule formation, changes in part of the fiber interval width, portal area and fibrous septum lymphocytic infiltration. (G) Laennec staging 4C, HE staining (4 x 10), hepatic lobule structure disappearance, liver cell vacuolization, focal necrosis, portal fibrosis formation of fibrous septa, fibrous septum wrapped around the liver cell cluster, formation of pseudolobules, fiber spacing width changes, portal area and fibrous septum lymphocytic infiltration).

Among the 396 liver tissue samples, there were 21 cases of L0, 40 of L1, 75 of L2, 89 of L3, 94 of L4A, 44 of L4B and 33 of L4C, as shown in Fig 2.

### The correlation between the serum HBV DNA level and liver Laennec staging

**Fig 3:**
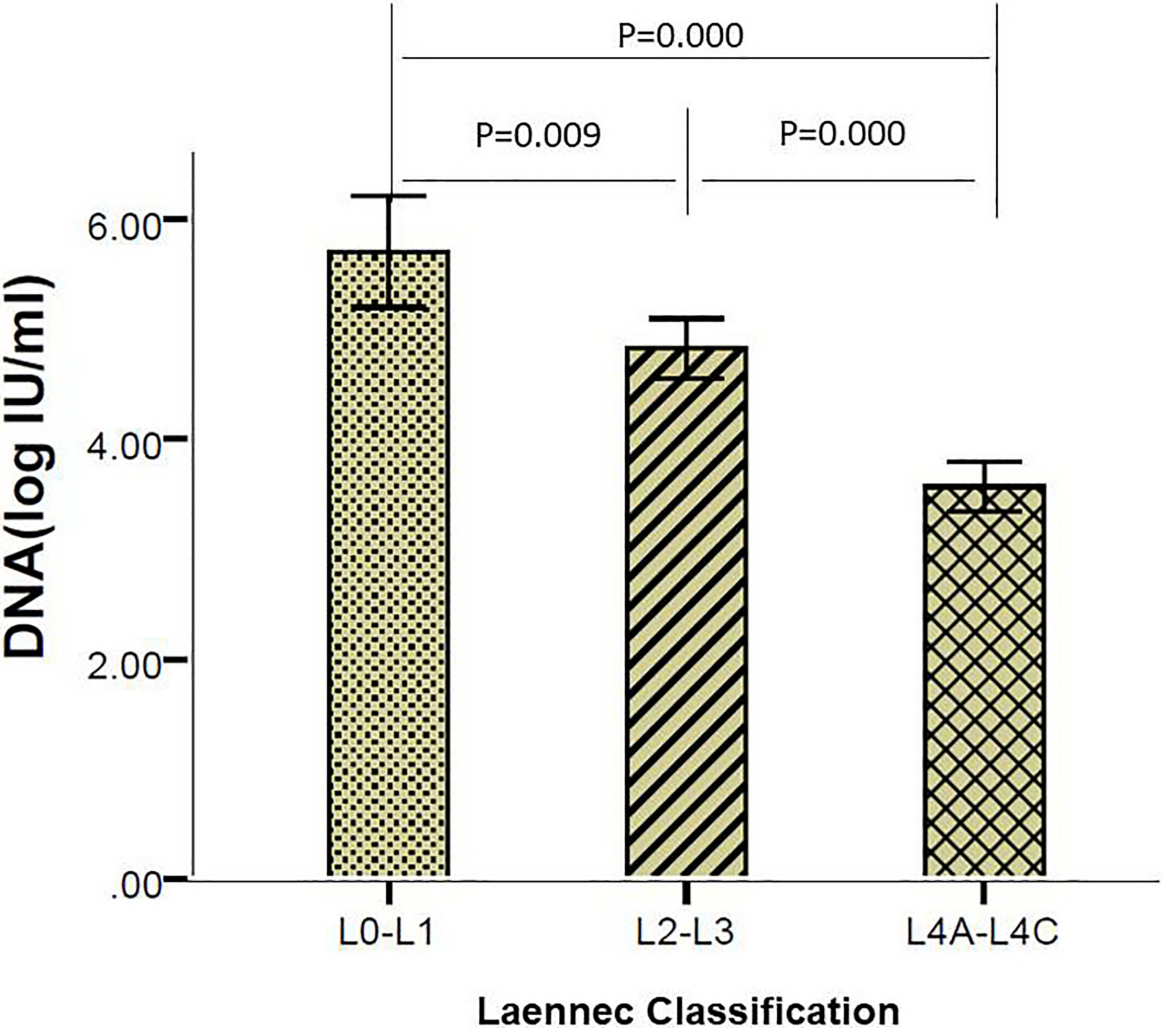
Comparison of the three Laennec grade groups and HBV DNA levels.

There were 61 cases of mild fibrosis (L0—L1), and the mean HBV DNA level was 5.71±1.98 log IU/ml. The moderate to severe fibrosis (L0—L1) group had 164 cases, and the mean HBV DNA level was 4.83±1.76 log IU/ml. There were 171 cases in the liver cirrhosis (L4A - L4C) group, and the HBV DNA level was 3.57±1.49 log IU/ml. The results showed that the pathological fibrosis of liver tissues increased with the decrease in HBV DNA level. There was a significant difference in the HBV DNA levels among the three groups (P = 0.009, P<0.001, P<0.001). The results are shown in Fig 3.

### The correlation between the serum HBV DNA level and liver Laennec staging of the HBeAg-positive group

The 396 patients were divided into two groups: a HBeAg-positive group and HBeAg-negative group; 151 (38.13%) cases were positive, and 245 (61.87%) cases were negative. There was a significant difference between the HBV DNA levels and the liver tissues with mild fibrosis, moderate to severe fibrosis and cirrhosis in the HBeAg-positive group (F = 17.585, P<0.001), and the HBeAg-negative group had the same results (F = 6.017, P = 0.003). The results are shown in Table 2.

**Table 2:** Comparison of the Laennec staging and HBeAg-positive HBV DNA in pathological liver tissues.

group	HBeAg positive		HBeAg negative	
	n (%)	HBV DNA (log IU/ml)	n (%)	HBV DNA (log IU/ml)
L0-1	42(27.81)	6.50±1.63	19(7.76)	3.95±1.50
L2-3	88(58.28)	5.32±1.72	76(31.02)	4.27±1.63
L4A-L4C	21(13.91)	3.88±1.64	150(61.22)	3.53±1.47
F		17.585		6.017
P value		< 0.001		0.003

### HBV DNA levels and liver fibrosis and cirrhosis in different groups

Cases were divided into three groups according to serum HBV DNA level. The results showed a significant difference in the stage of liver fibrosis and cirrhosis among the three groups (χ^2^ = 53.76, P<0.001). The results are shown in Table 3.

**Table 3:** Comparison of groups with different HBV DNA levels and liver fibrosis and cirrhosis.

HBV DNA groups	L0	L1	L2	L3	L4A	L4B	L4C	N
no replication	2	4	8	17	34	17	19	101
low replication	4	7	20	16	24	9	5	85
high replication	15	29	47	56	36	8	9	210
Total	21	40	75	89	94	44	33	396

χ^2^ = 53.76, P<0.001

### Logistic analysis of liver biopsy stages and clinical indexes

Liver biopsy samples were divided into three groups according to the severity of liver fibrosis cirrhosis: a mild fibrosis group (L0-L1), severe fibrosis group (L2-L3) and liver cirrhosis group (L4A-L4C). Multiple factors including sex, age, HBeAg state, HBV DNA quantitative logarithm, PLT and ALT level were analyzed by ordered multi-classification logistic regression. The results showed that HBV DNA was an independent predictor of the degree of liver fibrosis and cirrhosis (OR 0.778, 95% CI 0.697–0.869, P<0.001).

### Prediction of liver fibrosis and cirrhosis by HBV DNA cut-off values in patients with different HBeAg states

**Fig 4:**
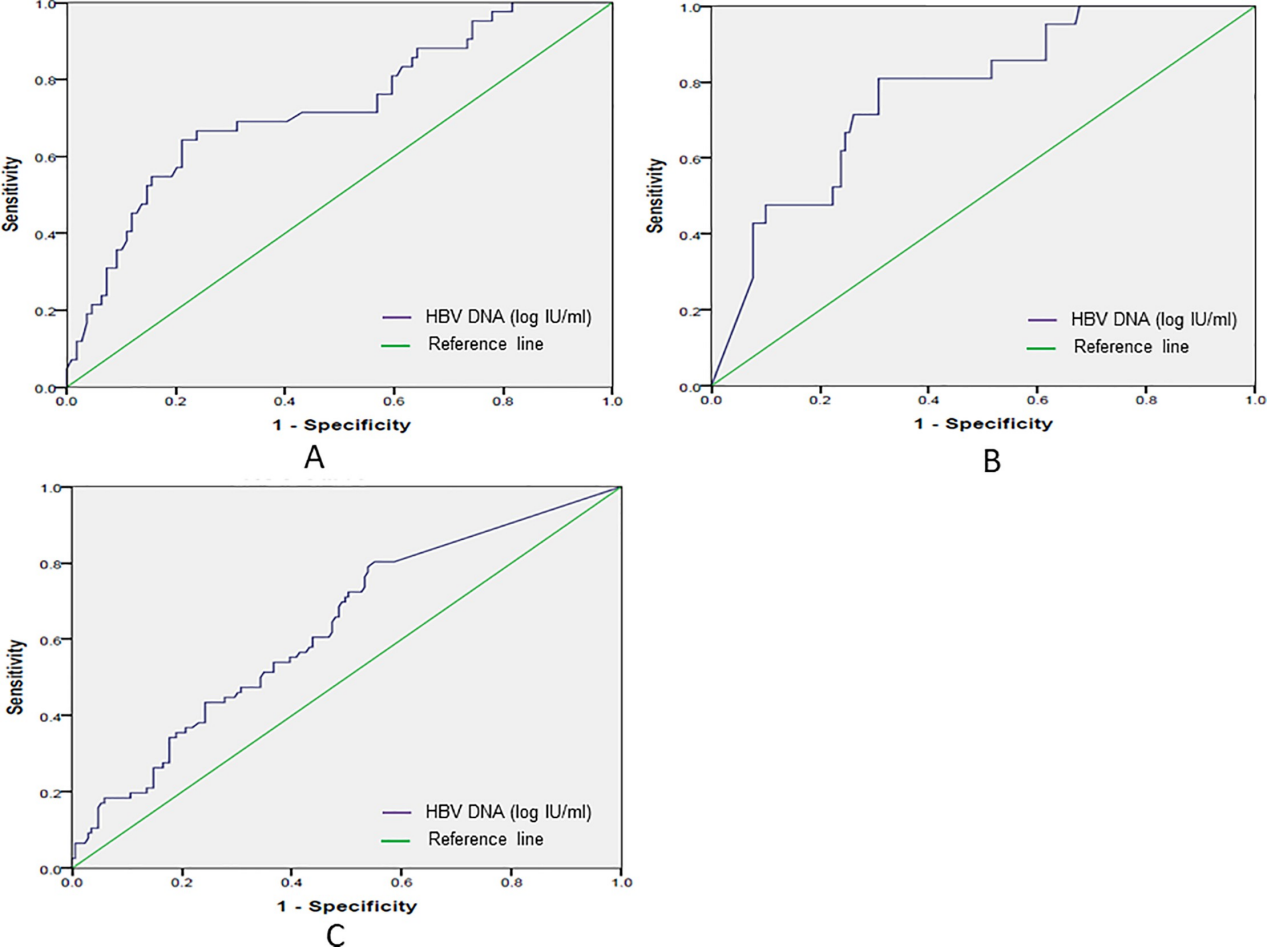
Prediction of liver fibrosis and cirrhosis by HBV DNA cut-off values. (A): L0-1 in HBeAg-positive patients. (B): L4A-4C in HBeAg-positive patients. (C): L4A-4C in HBeAg-negative patients).

The ROC curve analysis showed that the sensitivity and specificity of the prediction of mild liver fibrosis when the HBV DNA cut-off value was >6.68 log IU/ml were 64.3% and 78.94%, respectively, and the AUC value was 0.818 in the HBeAg-positive group. When the HBV DNA cut-off value was <4.94 log IU/ml, the sensitivity and specificity of the prediction of liver cirrhosis were 81.0% and 69.2%, respectively, and the AUC value was 0.871 in the same group. However, in the HBeAg-negative group, an HBV DNA cut-off value of <2.68 log IU/ml could predict liver cirrhosis with a sensitivity and specificity of 48.0% and 76.8%, respectively, and the AUC value was 0.697. The results are shown in Fig 4.

## Discussion

The effect of HBV DNA level on liver fibrosis and cirrhosis is a research hotspot [10–13]. However, the results and conclusions of previous studies have not been consistent [14,15]. The samples included in the studies were mostly patients with chronic hepatitis B [16,17]. Few studies have investigated liver cirrhosis samples. Most publications used the Ishak, METAVIR and Batts systems [18–20] to classify the degree of fibrosis in the liver tissue. There are relatively few studies on the relationship between HBV DNA levels and different stages of liver cirrhosis. The purpose of our research was to explore the correlation between these variables and provide a theoretical basis for treatment strategies and evaluation of prognosis of patients with hepatitis B and different HBV DNA levels who develop liver fibrosis or cirrhosis.

In this study, we used the Laennec histological staging system to divide the liver tissues with fibrosis or cirrhosis into 7 grades according to the thickness and quantities of hepatic interlobular septum: grades L0, L1, L2, L3, L4A, L4B and L4C. The results showed that the Laennec staging system, to some extent, could reflect the degree of liver cirrhosis, the clinical staging and the liver function and predict the incidence of complications in patients with cirrhosis [8,21,22].

The results of our research showed that the HBV DNA level decreased gradually with the severity of Laennec staging of the liver tissue. According to the degree of liver fibrosis and cirrhosis, we divided the liver samples into the mild fibrosis group (L0-L1), severe fibrosis group (L2-L3) and liver cirrhosis group (L4A-L4C). The differences in HBV DNA level among the three groups were significant. HBV DNA was an independent predictor of liver fibrosis staging. The result was consistent with the findings of Diktas H [16] and Surat P [23].

According to the Laennec staging system, the degree of liver tissue cirrhosis was divided into the L4A, L4B and L4C groups. We compared the HBV DNA levels among these three groups. The results showed that there was no significant difference between the L4A and L4B groups (Z = -0.260, P = 0.795), but the differences between L4A and L4C and between L4B and L4C were statistically significant (Z = -2.013, P = 0.041; Z = -1.961, P = 0.049). The HBV DNA level gradually decreased with the severity of liver tissue cirrhosis.

The results of Kim JD [5] suggested that patients in the late stage of liver cirrhosis (decompensation period) could still have significant inflammation of the portal area even if their serum alanine transaminase was normal or serum viral load was very low (<2000 copies/ml). The Oliveira VO study confirmed that low-load HBV DNA did not indicate that liver injury ceased; instead, the liver cell injury continued [24]. HBV DNA can still be detected in liver tissues of patients with liver cirrhosis without HBV DNA detection in the serum [25]. The studies of Chen JD and Fung J suggested that the pathological processes in liver tissues of inactive HBV carriers could show progression, and some may even develop into end-stage liver disease or hepatocellular carcinoma [26,27]. The continuous suppression or removal of the virus could reduce the progression of inflammation and fibrosis in the liver, preventing fibrosis or cirrhosis of the liver [28,29]. Our results showed that low levels of HBV DNA and the liver pathological changes were not parallel. Thus, the HBV DNA level may not reflect liver disease progression. For hepatitis B cirrhosis patients, anti-viral inclusion based only on the HBV DNA level or clinical diagnosis should be further studied.

In this study, 396 HBV-related liver disease patients were divided into the HBeAg-positive group and HBeAg-negative group. The HBV DNA levels showed significant differences among the mild fibrosis, severe fibrosis and cirrhosis cases in the HBeAg-positive group (F = 17.585, P<0.001), and the HBV DNA level decreased significantly with the severity in pathological changes of liver tissue. These findings were consistent with the studies of Xie Q [30] and Gao Y [31], who reported that extensive and repeated immune-mediated responses led to decreased viral load and liver damage. Our study suggested that the effects of HBV DNA level on the formation of liver fibrosis and cirrhosis might be enhanced in HBeAg-positive patients.

Many studies have reported that liver injury in patients with chronic hepatitis B was associated with HBV-mediated immune response rather than direct injury to hepatocytes [32,33]. The occurrence and development of liver fibrosis and cirrhosis might be caused by liver interstitial vascular chronic ischemia [34,35]. Chronic liver injury leads to endothelial cell injury and microvascular thrombosis, and then, cell proliferation and fibrosis gradually appear [36]. The degree of liver tissue injury may not have increased with the serum HBV DNA level, or there may be no parallel relationship between the two [1,37,38].

Our study results showed that the HBV DNA level decreased with the degree of liver fibrosis and cirrhosis. The replication of HBV DNA, a marker of HBV replication, depended on the liver cells, which may explain these findings. After infection by HBV, the body produces specific cellular and humoral immune responses that cause hepatocyte injury. With persistence of HBV infection, the cumulative immune damage increases gradually, and the extent of liver cell damage is also aggravated, causing extensive necrosis of the hepatocytes. Finally, hepatocytes are extensively necrotic, leading to a decrease in HBV, as replication is dependent on hepatocytes. Therefore, the more extensive the liver cell damage, the lower the HBV DNA load. We considered whether this process could similar to "seed growth dependent on the soil"; this hypothesis requires further study and discussion. Some scholars believe that the course of most patients with chronic hepatitis B is long. During this period, liver cell degeneration and necrosis occur repeatedly, and excess collagen deposition continues. All of these situations could lead to liver fibrosis or even cirrhosis. The study confirmed that the liver tissue injury and formation of liver fibrosis and cirrhosis had a significant correlation with the long-term infection of HBV DNA [39]. High replication of HBV DNA was an independent risk factor for the development of cirrhosis in chronic hepatitis B patients [40]. The continuous replication of HBV DNA in the liver cells resulted in the continuous aggravation of inflammatory necrosis and liver fibrosis. In patients infected by HBV, the degree of liver tissue inflammatory activity and fibrosis were significantly increased after repeated liver injury. Long-term HBV infection promoted the formation and severity of liver fibrosis and cirrhosis.

The etiology of liver disease in this study was only HBV, as other causes were excluded. The results of the study independently showed the influence of HBV DNA on liver fibrosis and sclerosis and their relationships. In this study, patients were divided into the HBeAg-positive group and the HBeAg-negative group according to HBeAg status so that we could evaluate liver fibrosis and sclerosis of patients infected by HBV at different stages.

Our study also has certain limitations. First, we lacked data on the HBV genotypes due to the limitations of our hospital's testing methods. Second, this study consists entirely of Chinese individuals in Hainan Province, who may have unique characteristics. Our results need to be confirmed by a multicenter study. The third limitation is that all patients included had not received regular antiviral treatment. With the current worldwide level of knowledge of hepatitis B, patients with chronic hepatitis B and cirrhosis who do not receive antiviral treatment are relatively rare, so the study is not repeatable.

This study results showed that the Laennec staging system could be used to classify the degree of liver fibrosis and cirrhosis according to the thickness and quantity of the interval. We investigated the relationship between the HBV DNA level and liver fibrosis and cirrhosis and provided one of the evidences supporting or discouraging antiviral therapy in patients at different stages. The relationship between HBV DNA level and liver fibrosis may suggest causality, and the results could provide support for antiviral therapy for patients at different stages. The study results suggested that the HBV DNA level decreased with the aggravation of fibrosis and cirrhosis of liver tissues. HBV DNA level was a good index for evaluating the degree of fibrosis and cirrhosis of patients with HBV-related liver diseases, especially HBeAg-positive patients.

## Supporting information

S1 Fig Flow diagram of the study population selection. (TIF)

S2 Fig Assessments of liver fibrosis and cirrhosis according to the Laennec staging system. (TIF)

S3 Fig Comparison of the three Laennec grade groups and HBV DNA levels. (TIF)

S4 Fig Prediction of liver fibrosis and cirrhosis by HBV DNA cut-off values. (TIF)

S1 Dataset Relevant data underlying the findings described in manuscript. (XLSX)
