# Supplementary figures and images for: The relationship between serum hepatitis B virus DNA level and liver histology in patients with chronic HBV infection
Source: PLoS One. 2018 Nov 7;13(11):e0206060. doi: 10.1371/journal.pone.0206060 (PMC6221304; doi:10.1371/journal.pone.0206060)

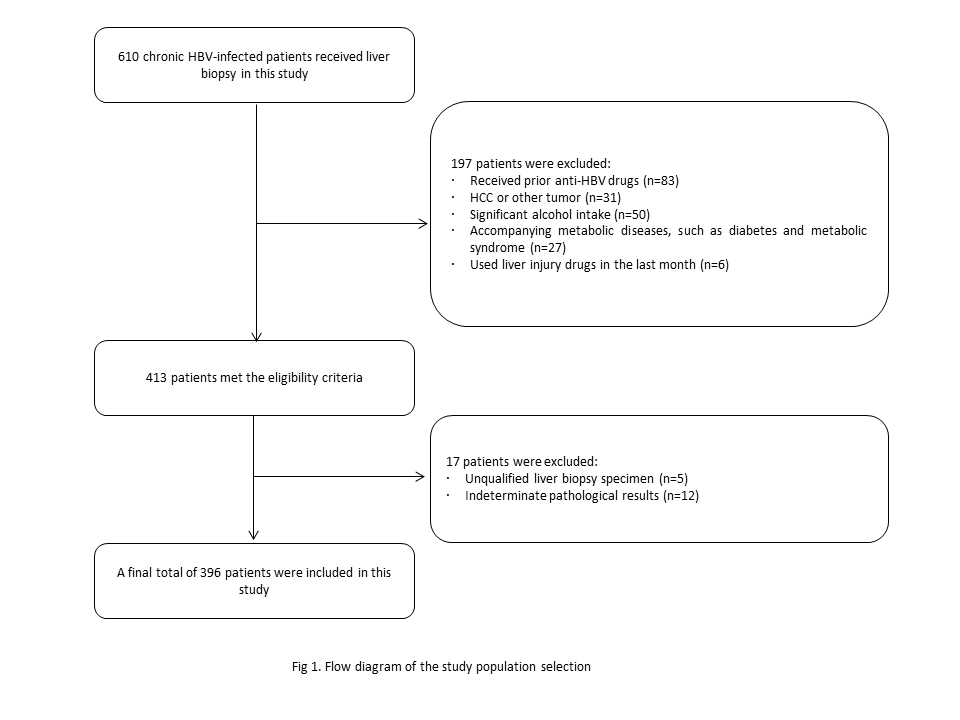

Supplement: S1 Fig — (TIF) [file pone.0206060.s001.tif]

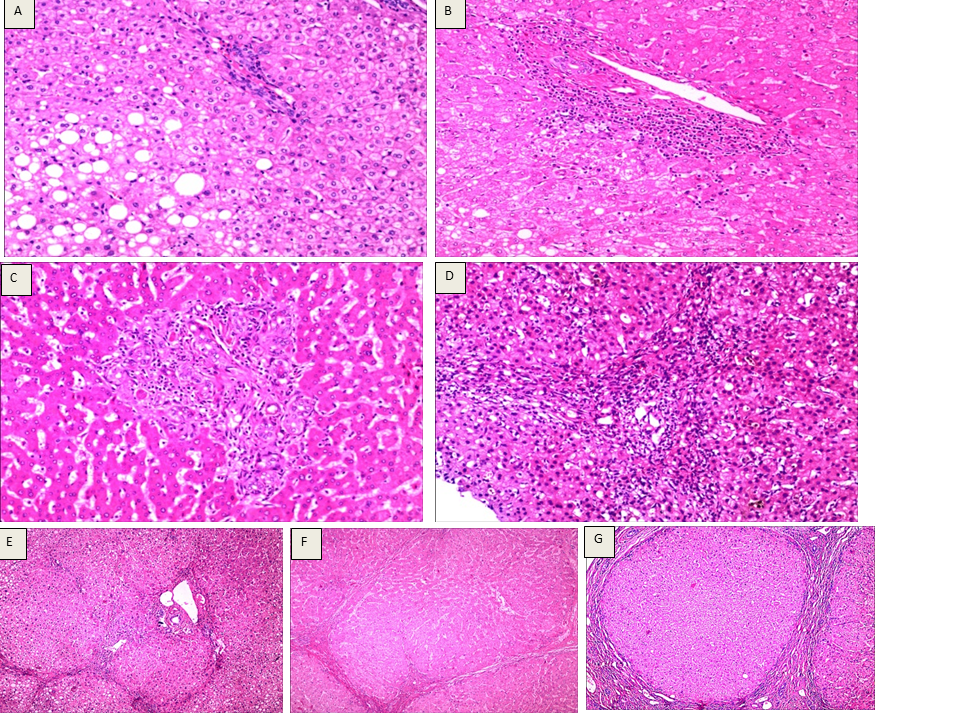

Supplement: S2 Fig — (TIF) [file pone.0206060.s002.tif]

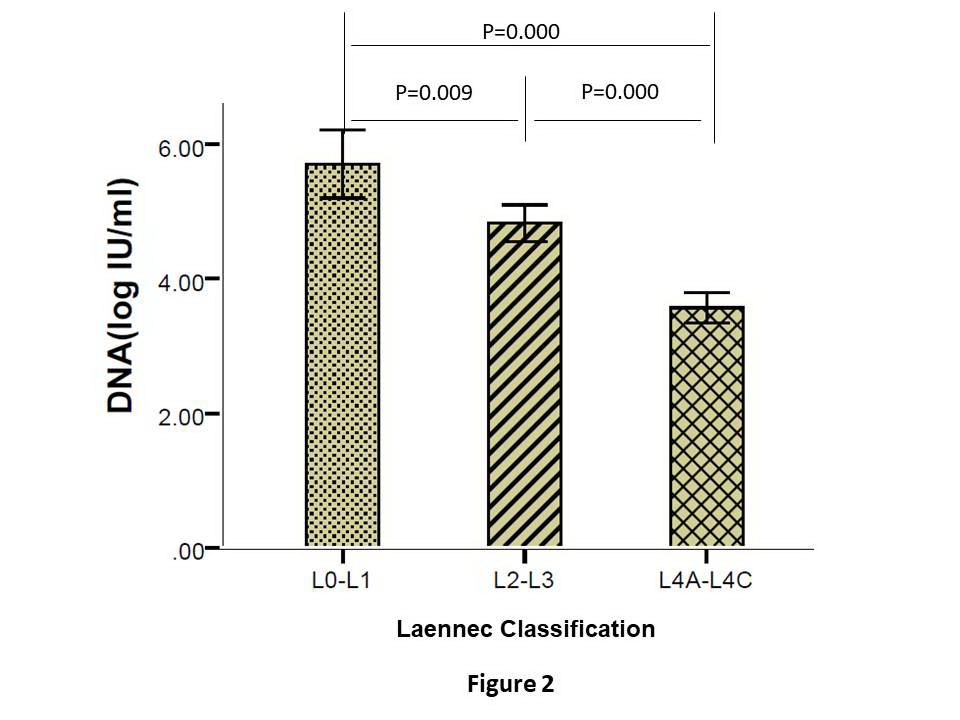

Supplement: S3 Fig — (TIF) [file pone.0206060.s003.tif]

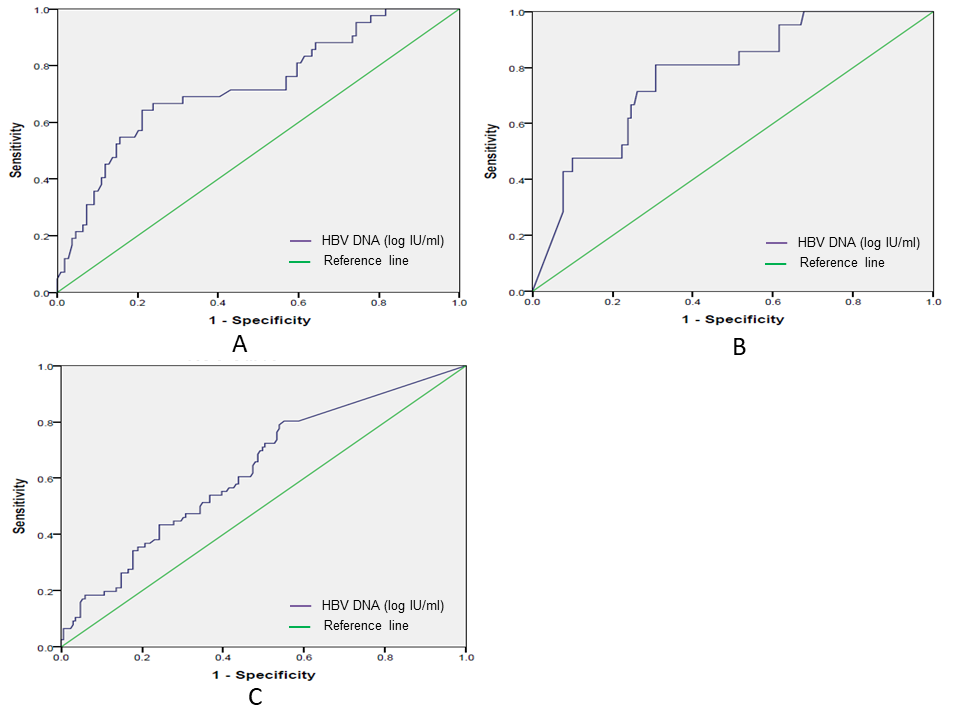

Supplement: S4 Fig — (TIF) [file pone.0206060.s004.tif]
